# Effect of Content and Surface Modification of Fique Fibers on the Properties of a Low-Density Polyethylene (LDPE)-Al/Fique Composite

**DOI:** 10.3390/polym10101050

**Published:** 2018-09-20

**Authors:** Mario Fernando Muñoz-Vélez, Miguel Angel Hidalgo-Salazar, Jose Herminsul Mina-Hernández

**Affiliations:** 1 Research Group for Manufacturing Technologies GITEM, Universidad Autónoma de Occidente, 760030 Cali, Colombia; mfmunoz@uao.edu.co (M.F.M.-V.); mahidalgo@uao.edu.co (M.A.H.-S.); 2 Composite Materials Group, Universidad del Valle, 76001 Cali, Colombia

**Keywords:** composite, LDPE, natural fibers, physico-mechanical properties, thermal properties, surface modification

## Abstract

This work presents the physical-thermal and mechanical characterization of a low-density polyethylene (LDPE)-Al matrix composite material that was obtained from reinforcing recycled (post-consumer) long-life Tetra Pak packages with fique natural fibers from southwestern Colombia. The fique was subjected to three chemical treatments to modify its surface (alkalinization, silanization and pre-impregnation with polyethylene) to increase the quality of its interfaces. Additionally, panels with 10%, 20%, and 30% *v*/*v* of fiber were manufactured by the hot compression molding. The mechanical properties of the different composite materials showed that the pre-impregnation treatment promoted a significant increase in the tensile and flexural properties with respect to the fiber-reinforced composite without surface modification. Additionally, in materials with 30% fibers that were treated with pre-impregnation, there was a decrease in the water absorption capacity of 53.15% when compared to composites made with 30% native fibers. Finally, increases in the fiber content mainly caused better mechanical performances, which increased as a direct function of the amount of fique incorporated.

## 1. Introduction

Composites are materials that have aroused great industrial interest due to their good properties. Their physical-mechanical performance depends on the properties and characteristics of each of the phases that compose them, the fiber arrangement in the composite, and the interfacial quality [1,2].

Among the materials that are currently used for the reinforcement of polymer matrices, natural fibers offer better mechanical properties than many of the polymers used as matrices in composites. However, natural fiber-reinforced thermoplastic matrix composites present some difficulties, which is mainly due to poor compatibility between the phases, i.e., the hydrophilic nature of the fibers and the hydrophobic characteristics of the matrix. Therefore, several investigations have studied the effect of different chemical treatments on the reinforcement material to improve the quality of the interface between the fibers and the matrix and improve the mechanical performance [3]. Among cellulosic fibers, fique is the fiber with the largest production in Colombia and it is grown in different regions of the country, mainly in Cauca, Nariño, Santander, and Antioquia, which produce 98% of the fiber in the country [1,4].

Additionally, the past few years have seen an increase in the use of thermoplastic polymers from recycled products with high volumes, such as the low-density polyethylene (LDPE)-Al that was obtained from recycling of postconsumer long-life Tetra Pak packaging. These materials are composed of 5% aluminum, 20% polyethylene, and 75% paper, materials with a very long decomposition period. The recycling of this product is based on the separation of the cellulosic pulp from the polyethylene and aluminum layers that compose the material in a process called hydropulping. Tetra Pak residue has been used worldwide for the manufacture of chipboard, from which different products are manufactured, such as plastic tiles, roofing sheets, and desks [1,5,6,7].

Due to the large production of fique in Colombia, there is interest in reusing the LDPE-Al from post-consumer Tetra Pak packages and in providing LDPE-Al with properties that allow for it to be used in products of greater structural demand than can currently be manufactured. In this study, the influence of the incorporation of fique fibers to reinforce an LDPE-Al matrix and improve its physical-thermal and mechanical properties was studied. Additionally, the effects of the surface modification of these fique fibers on these properties were studied.

Samples of the composite materials were fabricated and evaluated based on the application of a 4 × 4 factorial design, in which the study factors were the surface treatment of the fibers at four levels (without treatment, alkalinized, alkalinized + silanized and alkalized + silanized + pre-impregnated with polyethylene), and the content of fibers to be incorporated in the composite (0, 10%, 20%, and 30% by volume). Additionally, some mechanical properties of the composite were studied, such as the tensile and flexural strength and the modulus. Finally, other physical-chemical properties, such as the density, thermal stability, percentage of crystallinity, and water absorption, were evaluated.

## 2. Materials and Methods

### 2.1. Materials

In this study, LDPE-Al that was obtained from the recycling (post-consumer) of long-life Tetra Pak packaging was used as the matrix of the composite; this residue was processed to obtain a particle size that increased the wettability of the fibers, a better distribution thereof in the matrix, and a direct contact between the phases composing the material. LDPE-Al had an LDPE:Aluminum volume ratio of 89.75:10.25 and a density of 1.10 g/cm^3^. The fique fibers used as a reinforcement belonging to the (white) eagle claw variety of the Furcraea genus and were supplied by Compañía de Empaques SA from Medellín (Medellin Packing Company SA), Colombia [8]. These fibers had an average length of 51.70 ± 33 mm (aspect ratio specified in Table 1) and a density of 1.10 g/cm^3^.

To prepare the composite boards, the fibers were arranged in a random, two-dimensional distribution. The densities of the fibers after the treatments varied due to the removal and/or addition of components therein (Table 1) [9]. In the alkalinization treatment of the fibers, sodium hydroxide reagent grade from Merck Corporation (Darmstadt, Germany), was used. Likewise, in the silanization of the fibers, Tris(2-methoxyethoxy)(vinyl)silane (“A-172” from Sigma Aldrich, St. Louis, MO, USA) and dicumyl peroxide were used as the coupling agents, and acetic acid was used to adjust the pH of the water-methanol solution; these latter reagents were purchased from Merck Corporation (Darmstadt, Germany). Finally, in the pre-impregnation treatment, xylene, reagent grade from Merck Corporation, was used to dilute the LDPE from INEOS Olefins & Polymers (Long Beach, CA, USA), which has a melt flow index of 7.50 g/10 min, a density of 0.92 g/cm^3^, and a melting temperature of 108 °C.

### 2.2. Surface Treatment of the Fibers

To increase the interfacial adhesion in the LDPE-Al/fique composite, the fibers were surface modified with three chemical treatments (Table 1): alkaline solution treatment (Fique-A), silanization (Fique-AS) and pre-impregnation (Fique-ASP); and, the methodology used for each treatment is described below:

#### 2.2.1. Treatment with Alkaline Solution (Fique-A)

Fique fibers were subjected to an alkalinization treatment to superficially remove hemicellulose and lignin, as well as external substances that could have formed during their production [10]. The treatment was performed in a similar manner to that reported for henequen fibers [11]. Here, the fique fibers were immersed in 2% *w*/*v* aqueous sodium hydroxide solution (NaOH) for one hour at 25 °C, and then the fibers were washed with distilled water until a neutral pH (6.5–7.5) was reached (the NaOH was completely removed). Finally, the filaments were subjected to a drying process, which was performed in two stages: the fibers were dried first at room temperature for 12 h and then in an oven at 60 °C for 24 h.

#### 2.2.2. Silane Treatment (Fique-AS)

Once the surface was treated with NaOH, the fibers were grafted with a coupling agent, Tris(2-methoxyethoxy)(vinyl) silane (“A-172”) to promote the formation of primary bonds between the silanized fibers and the LDPE-Al matrix. The treatment was performed in a similar manner to that reported by Valadez et al. [3]. Fique fibers were immersed in a 50/50 *v*/*v* water-methanol solution, in which 1% and 0.50% of silane and dicumyl peroxide, respectively, had been previously dispersed. The pH of the solution was adjusted to 3.5, and the solution was stirred for 30 min. The fibers were then immersed for 1 h, decanted and then dried for a period of 24 h at 60 °C. Finally, the fibers were heat cured for 2 h at 120 °C.

#### 2.2.3. Pre-Impregnation Treatment (Fique-ASP)

Finally, fique fibers that were already modified with the surface alkalization and silanization treatments (Fique-AS) were pre-impregnated with a polyethylene solution. This surface modification was conducted to facilitate the impregnation and wetting of the fibers with the LDPE-Al matrix during the compositing process. The conditions of this treatment accounted for the process parameters that were established for the treatment of henequen fibers [11]. Fique fibers were immersed in a 1.5% LDPE-Xylene solution (*w*/*w*). The pre-impregnation treatment was conducted at 120 °C and a constant stirring speed of 100 rpm for 1 h. Then, the fibers were dried at 60 °C for 24 h. For all of the surface treatments, 300 mL of solution per 25 g of fique fibers was used.

### 2.3. Preparation of the Composite and Test Pieces

**Figure 1:**
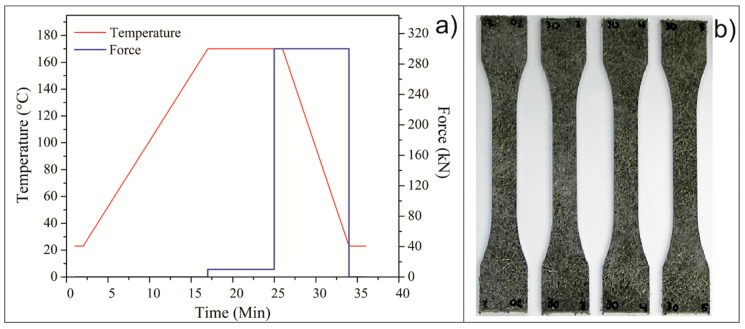
(**a**) Manufacturing process cycle, and (**b**) appearance of specimens after the process of routing and sanding.

LDPE-Al/fique boards with dimensions of 290 × 290 × 2 mm were produced while using a stainless steel frame-type mold. The panels were fabricated by a compression molding process with hot plates in a LabPro400 press (Boca Raton, FL, USA) from Fontijne Presses. The parameters that were used in the process are shown in Figure 1. The processing temperature was 170 °C, and the force exerted by the plates was 300 kN, which provided a pressure of approximately 3 MPa. Boards were made with 10%, 20%, and 30% fique fibers (with or without treatments) by volume.

Then, the test specimens were manufactured while using numerical control machining with a CNC router and finally the edges were sanded to eliminate the edge effects (Figure 1b). The specimens were prepared according to the dimensional parameters established in the ASTM D638 and D790 standards.

### 2.4. Mechanical Characterization

The tensile and flexural properties of the LDPE-Al/fique composites with different fiber content and different surface treatments were determined. These tests were conducted while using a universal test machine INSTRON 3366 (Instron, Norwood, MA, USA) with a load cell of 10 kN. The velocity used in the tensile tests was 5 mm/min, whereas in the flexure tests, the velocity was determined as a function of the dimensions of the specimen (within the range of 0.79–1.01 mm/min). The test specimen “Type I” was used for the tensile tests (ASTM D638). In flexion (ASTM D790), the distance between supports was defined as 16 times the thickness of the test sample, and the dimensions of the specimens were 12.7 × 100 × thickness (which varied between 1.83 and 2.37 mm, depending on the composite). The test was performed until the sample reached 5% deformation. Five specimens of each of the composites were evaluated for each test.

### 2.5. Physical and Thermal Characterization

#### 2.5.1. Density

This test was performed according to the Archimedes method that was proposed in the ASTM D792 standard. Due to the hydrophilicity of the fique fiber, the conventional density test of immersion in distilled water was discarded, and canola oil (density of 0.91 g/cm^3^) was used instead as the immersion liquid. To compare the experimental results with theoretical values, the density of each of the evaluated composites was determined while using the Equation (1), rule of mixtures of a perfect composite (without voids), which gave density values of the fique fibers after different surface modifications (Table 1).

(1) $$\rho_{c}=\rho_{f}V_{f}+\rho_{m}\left(1-V_{f}\right)$$

where *ρ* is the density and the subindices c, f, and m, refer to the composite, the fiber, and the matrix, respectively; and, *V*_f_ is the volume fraction of fiber incorporated into the composite.

#### 2.5.2. Water Absorption (ASTM D750)

The influence of the fiber content (10%, 20% and 30%) and type of treatment it received (Fique-N, Fique-A, Fique-AS and Fique-ASP) on the ability of the composite to absorb water was studied.

The test was carried out according to ASTM D570, in a Memmert water bath equipment. First, the materials were conditioned in an oven for 24 h at 50 °C, after the conditioning time, the specimens were weighed (conditioned weight). Afterwards, the specimens were immersed for 24 h in distilled water at 25 °C. Finally, they were removed from the water, dried superficially, and weighed (wet weight); the determination of the percentage of water absorbed was made according to the model specified in Equation (2).

(2) $$\mathrm{Water}\;\mathrm{absorption}\;\left(\%\right)=\frac{\mathrm{wet}\;\mathrm{weight}-\mathrm{conditioned}\;\mathrm{weight}}{\mathrm{conditioned}\;\mathrm{weight}}$$

#### 2.5.3. Thermogravimetric Analysis (TGA)

The thermal stability of the materials under study was determined while using a TGA Q500 Thermogravimetric Analyzer from TA Instruments (New Castle, DE, USA) with a nitrogen protective atmosphere at a heating rate of 10 °C/min and a temperature range of 25 to 550 °C. The amount of sample used in the test was approximately 10 mg.

#### 2.5.4. Differential Scanning Calorimetry (DSC)

Thermal transitions and crystallinity in the composites were determined while using a DSP Q2000 DSC Differential Scanning Calorimeter from TA Instruments under a protective nitrogen atmosphere at a heating and cooling rate of 10 °C/min. Three stages were performed: (I) heating from −90 to 150 °C and an isotherm at 150 °C for 4 min, (II) cooling from 150 to −90 °C and an isotherm at −90 °C for 3 min, and (III) heating from −90 to 150 °C. This technique allowed for the determination of the melt temperature of the LDPE-Al matrix and the influence of the incorporation of fique fibers on the crystallinity of the polyethylene phase of the composite. The percentage of crystallinity was estimated while using Equation (3).

(3) $$X=\frac{\Delta H_{f}}{W_{\mathrm{pe}}\;\Delta H_{f}^{0}}\times 100$$

where X is the percentage of crystallinity, ∆*H*_f_ is the melting enthalpy of the composite, $\Delta H_{f}^{0}$ is the melting enthalpy corresponding to 100% crystalline polyethylene (288.83 J/g), and *W*_pe_ is the mass fraction of the polyethylene phase in the material [12].

## 3. Results and Discussion

### 3.1. Tensile Properties of the Composites

**Figure 2:**
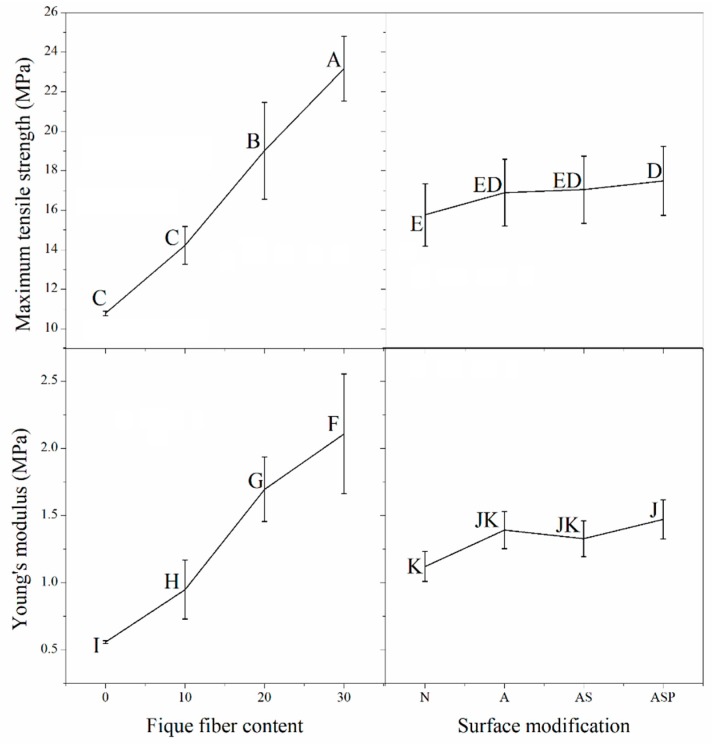
Principal effects for the maximum tensile strength and the Young’s modulus (According to the statistical analysis the values that do not share a letter are significantly different).

A statistical analysis was performed on the 4 × 4 factorial model to study two response variables: the maximum tensile strength and the Young’s modulus. It was found that surface modification and fiber content act independently on these response variables. Thus, the effects of the factors were studied independently (Figure 2).

The effects of the different levels of the “surface modification” factor on the maximum tensile strength and the Young’s modulus of the composite were investigated. The effects were statistically the same when the material was reinforced with fibers that had been subjected to an alkaline treatment (i.e., Fique-A, Fique-AS, and Fique-ASP). However, the ASP treatment gave better performance than the untreated fiber in these two response variables. The best performance was observed from the composites that were reinforced with the Fique-ASP fiber (Figure 2), which obtained 17.45 MPa and 1.47 GPa for the maximum tensile strength and the Young’s modulus, respectively. The above could be attributed to the fact that the pre-impregnation treatment promotes the wettability of the fibers with the polymer, which increases the interdiffusion of the polymer phase and thus the interactions between the fibers and the matrix. Additionally, mechanical anchoring interactions, which predominate in composites made from fiber mats, increase because of the high aspect ratios of the fibers and their arrangement [3,8,13,14].

The effects produced by the “fiber content” factor on the response variables differed between the 10%, 20%, and 30%, with 30% fique fiber content (regardless of the type of fiber used) having the greatest maximum strength (23.29 MPa) and the highest Young’s modulus (2.10 GPa). These values were similar to those that were reported by Valadez-González et al. [3], who reported strengths between 21 and 27 MPa for HDPE composites and similarly modified henequen fibers in samples with 20% *v*/*v* of fibers that were 6 mm in length. In addition, a 10% fiber content in the composite changed the Young’s modulus from that obtained in the composite without fibers, whereas no significant effect was observed for the maximum tensile strength between the sample with 10% of fiber and the composite without fiber. This can be attributed to the fact that the composite acquired part of the stiffness of the fique fibers, which prevented the large deformations that usually occurs in LDPE from recycled Tetra Pak packages. The recycled polyethylene has a deformation value of approximately 92%, while fique fibers present deformations of approximately 11% [9,15].

### 3.2. Flexural Properties of the Composites

**Figure 3:**
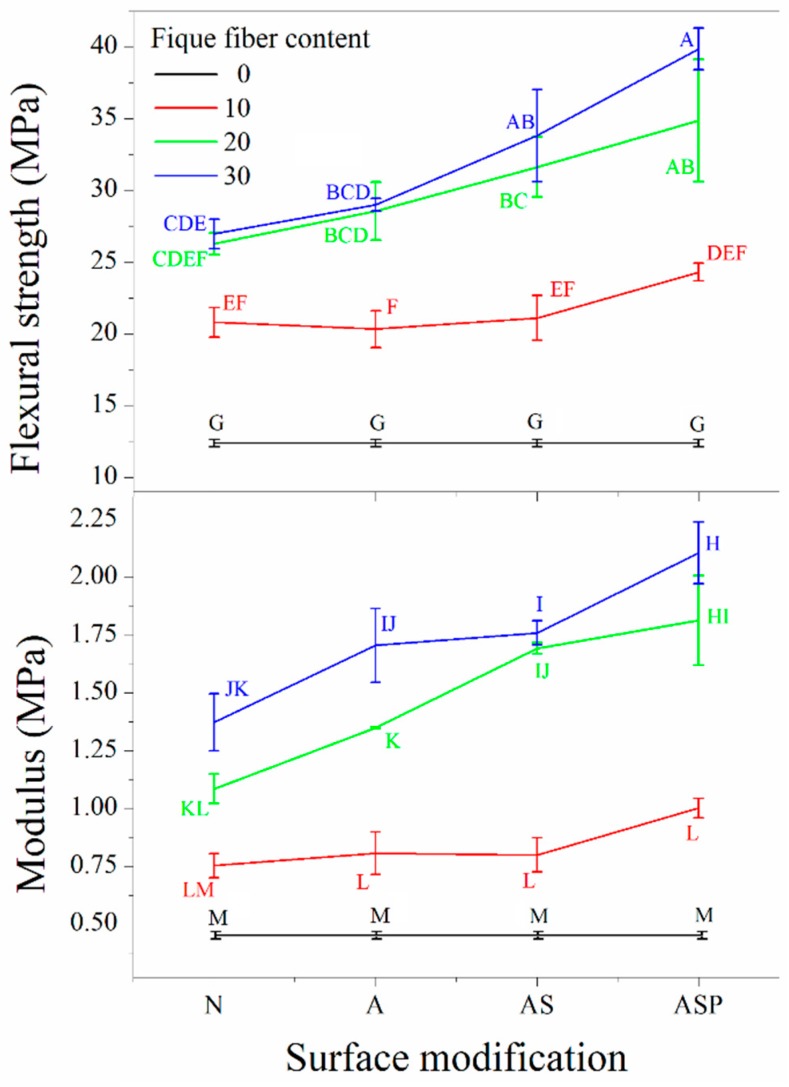
Principal effects on the maximum flexural strength and the flexural modulus (according to the statistical analysis the values that do not share a letter are significantly different).

A statistical analysis established a dependence between the two factors, fiber content and surface modification. Accordingly, we analyzed the effects of the factors together. To identify the influence of the factors and their levels on the response variables, in addition to comparing the different composites, a post-ANOVA test was performed, the results of which are shown in Figure 3.

From the results that were obtained in the post-ANOVA test, it was possible to conclude that as the amount of fibers in the composite increased, the maximum strength and the modulus of the material increased. This effect could be observed in any of the LDPE-Al/fique composites evaluated, regardless of the surface modification technique used on the fibers. In LDPE-Al/fique samples with 20% and 30% fique, the flexural mechanical properties increased with the treatments in the following order: Fique-N < Fique-A < Fique-AS < Fique-ASP, whereas in composites with 10% fiber, a significant increase was observed in maximum strength and the modulus only when reinforcing the composite with Fiber-ASP, this in comparison with Fiber-N composites.

The above could be attributed to the effect of the chemical treatments used, which promoted various mechanisms of interfacial adhesion between the fibers and the matrix. The nature of these treatments became more important at high fiber contents because the probability of forming such fiber-matrix adherence mechanisms increased. However, the low flexural mechanical properties of Fiber-N-reinforced composites were due to the poor compatibility between the hydrophilic (polar) fibers and the hydrophobic (apolar) polymer matrix. The tendency of the maximum strength and the modulus to increase as more surface chemical treatments were used on the fique fibers was due to (I) an increase in the mechanical adhesion that is promoted by the generation of greater surface area and roughness on the fiber as a consequence of the treatment in an alkaline solution [15]; (II) the formation of covalent bonds with the silane coupling agent in the fiber silanization treatment, specifically through of the silicofunctional group with the hydroxyl groups (OH) of the fiber and the organofunctional group with the polymer [9,16]; and, (III) the increase in the compatibility between the fiber and the matrix due to the pre-impregnation treatment of the fibers, which decreased the fibers’ polarity to better match the typically apolar thermoplastic matrices [9,17].

Due to these effects and to the different interfacial adhesion mechanisms that are promoted by the NaOH, silane, and pre-impregnated treatments, the material reinforced with 30% Fique-ASP fibers showed the largest maximum strength and flexural modulus: 39.90 MPa and 2.11 GPa, respectively. Importantly, these results were higher than those that were reported by Hidalgo et al. [2], who reinforced an LDPE-Al matrix with 30% fique fibers to obtain 31.26 MPa for the maximum strength and 1.48 GPa for the flexural modulus.

### 3.3. Density

**Figure 4:**
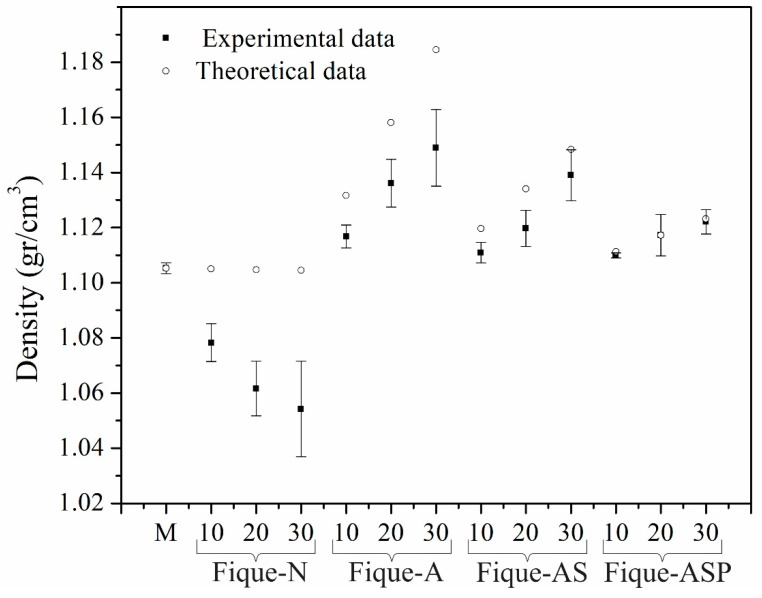
Density of the low-density polyethylene (LDPE)-Al and composites with 10%, 20%, and 30% fique fiber with or without surface modification.

The densities of the different fabricated composites and the LDPE-Al matrix were estimated to establish the influence of the fiber content and the surface modification on the density of the material. Figure 4 shows the obtained results and the density values that were estimated while using the rule of mixtures (Equation (1)).

The density of the matrix decreased as the content of N-Fique fibers in the material increased. This occurred even though it was expected (according to the theoretical data) that the density of the composite would not vary substantially with respect to the density of the matrix. This effect of the N-Fiber content on the density of the material can be attributed to the formation of a weak interface between the phases of the composite, which could have had an effect on the generation of voids and/or cavities between the fiber and the matrix. Thus, the greater the amount of fiber in the material is, the greater the difference between the theoretical and experimental values.

In composites made with Fique-A fibers, the experimental results followed a trend similar to that estimated from the theoretical values. However, the recorded values were inferior to the theoretical ones, and the experimental error increased with increases in the fiber content, which led to the conclusion that the interface between the fiber and the matrix still allowed for the formation of voids and/or cavities between the phases of the composite. However, this difference is less marked than that shown by materials with untreated fibers.

The results for materials reinforced with Fique-AS fibers showed that the difference between the experimental and theoretical data was approximately constant across different fiber contents. Therefore, it was possible to conclude that the increase in the content of this type of fiber did not generate an increase in the number of voids in the material. In turn, the composites with Fique-ASP fibers had densities that were very similar to those estimated by the rule of mixtures. Thus, the silanization and pre-impregnation treatments improved the quality of the fiber-matrix interface, which, in turn, decreased the number of cavities between the phases. This occurred because the silane coupling agent promoted chemical bonds (between the fique and the LDPE material) and the pre-impregnation treatment allowed for greater wetting of the fiber with polyethylene.

### 3.4. Water Absorption

**Figure 5:**
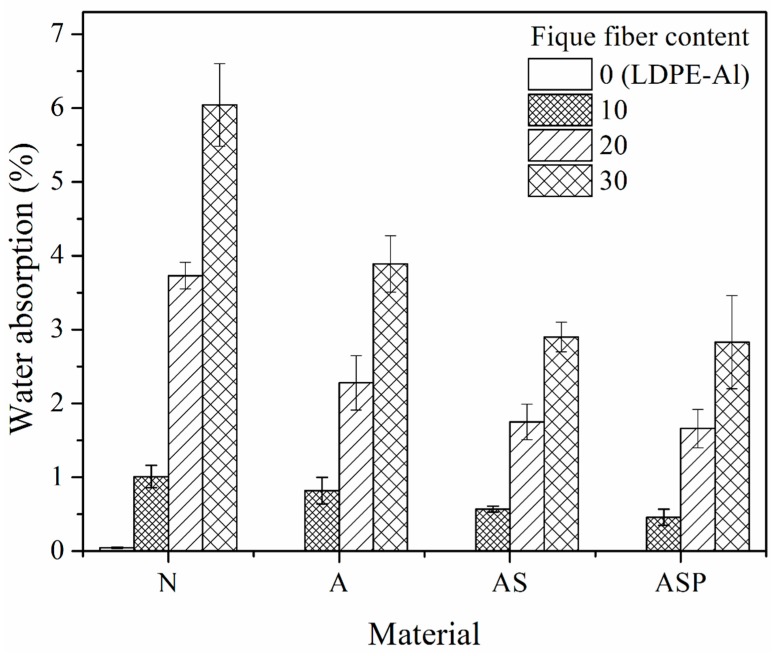
Absorption of water by immersion at 23 °C for the composite materials reinforced with fique fiber with or without surface modification.

Due the properties of the composite could be affected by the absorption of water promoted by the incorporation of hydrophilic fibers into the material, the influence of the percentage of fique fibers (with different surface treatments) on the capacity of the material to absorb water was determined [18]. Figure 5 shows that the non-reinforced LDPE-Al matrix had a water absorption percentage of 0.05%, which increased with the content of incorporated fibers. On the other hand, materials reinforced with Fique-N fibers showed a higher tendency to capture water when compared to composites made with fibers modified with NaOH, regardless of the fiber content. This phenomenon can be attributed to the hydrophilic nature of the cellulosic fibers, especially hemicellulose, which is the component with the greatest capacity to absorb water from the fiber wall due to its disorganized intermolecular structure, which makes it difficult for its hydroxyl groups to establish intermolecular hydrogen bonds [19]. According to the above, the increased weight from the absorption of water in the composites with Fique-A fibers could have resulted from diffusion and percolation. The latter is a phenomenon that is generated by micro-spaces present between the fiber and matrix that allow for the filtration of water through the material. This phenomenon becomes more important as the content of the fibers in the matrix increases. However, by removing the hemicellulose by treatment with an alkaline solution, the hydrophilic character of the natural fibers is limited to the amorphous regions of the cellulose because, in the crystalline regions, the hydroxyl groups from neighboring chains of cellulose bond with each other. Additionally, lignin has been determined to be a polymer with the lowest capacity to absorb water from the fiber [19,20,21].

At the same time, composites that were treated with NaOH + silane (Fiber-AS and Fiber-ASP) absorbed less water than those treated with only NaOH. This can be associated in part with the fact that the silanization and pre-impregnation treatments improved the interfacial quality of these composites, reducing the percentage of cavities and/or voids that can promote percolation. Additionally, during the silanization treatment, some OH groups of the cellulose interact with the silane by establishing hydrogen bonds; therefore, the number of interaction points of the water molecules with the hydroxyl groups residing in the cellulose was reduced, which reduced the ability of the fibers to capture water [16,22]. Importantly, the pre-impregnation treatment covers the fique fibers with a thin layer of polymer that hinders interactions between the ambient water and the surface of the naturally hydrophilic fiber.

In addition to decrease in the diffusion and percolation effects with natural fiber surface treatments, it is important to note that the hydrophobicity could have increased in the treated fibers. Muñoz et al., in 2014 found that a natural fique fiber has a contact angle (with respect to water drop) of approximately 57.28°, while a fique fiber that was treated with alkalization + silanization, showed a contact angle of approximately 71°. In the same way they observed that on fique fiber treated with alkalization + silanization + pre-impregnation, the contact angle increased to 86.66°, which corroborates the increase in the hydrophobicity of the fibers with the AS and ASP treatments [9].

### 3.5. Thermogravimetric Analysis (TGA)

**Figure 6:**
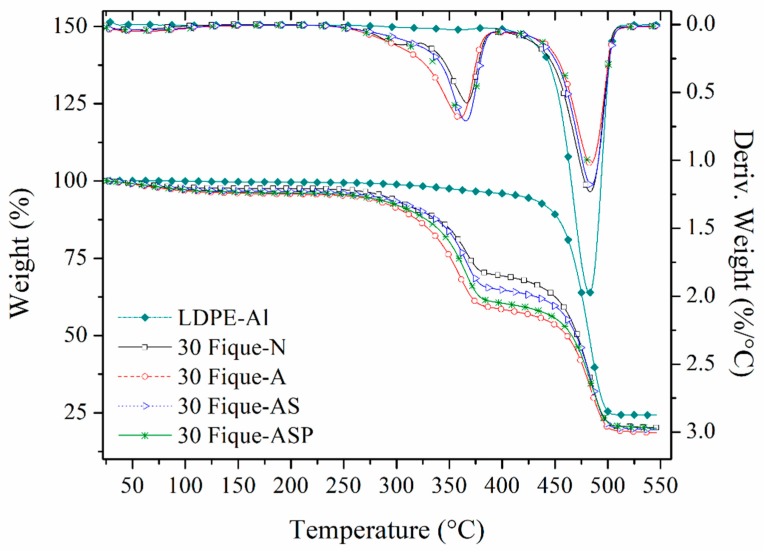
Thermogravimetric Analysis (TGA) thermogram for LDPE-Al and composites with 30% fique fiber with different surface modifications.

The mechanical properties of the composites also depend on the working temperature, so it is important to know the thermal stability of the materials that were studied. A thermogravimetric analysis was conducted on the composites with 30% fique fibers by volume that underwent the different surface treatments (Fique-N, Fique-A, Fique-AS, and Fique-ASP), since they showed the best mechanical properties. Additionally, the unreinforced LDPE-Al matrix was evaluated for comparison purposes (Figure 6).

In the thermogram of LDPE-Al (M), the highest mass loss rate of the polyethylene occurred at approximately 470 °C, reaching a material degradation of 75% at this point. Because polyethylene residues are not significant at this temperature (0.330%) [22], the above suggests that the remaining 25% of the material corresponded to the aluminum, which, at these temperatures, remains solid. This LDPE/Al 75/25 mass ratio is equivalent to a ratio of 89.75/10.25 by volume.

As for the materials that were reinforced with 30% fiber, it was possible to observe two thermal events associated with the fique fibers (degradation of hemicellulose and cellulose) before the degradation of the LDPE [23]. Particularly, in the composite with untreated fibers, an additional mass loss stage was observed at 305 °C (attributed to the degradation of the hemicellulose), which was not present in the composites with fibers treated with NaOH. Additionally, the peak corresponding to the temperature of the greatest mass loss in the LDPE was shifted from approximately 470 to 483 °C in all of the reinforced materials. This increased thermal stability has been reported in several other investigations [12,24,25], which indicates that the incorporation of the fibers into the material introduces nucleation points that increase the crystallinity of the polymer, and, in turn, improve its thermal properties.

### 3.6. Differential Scanning Calorimetry (DSC)

**Figure 7:**
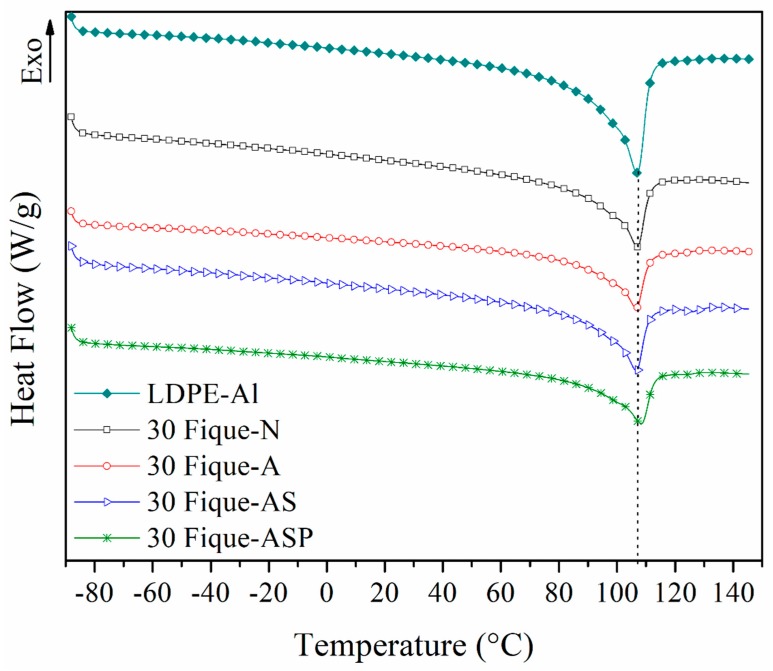
Heat flow vs temperature for LDPE-Al and composites with 30% fique fibers with different surface modifications (second heating).

The thermal transition corresponding to the melting of the LDPE phase of the composite materials reinforced with 30% fique fibers (with and without treatment) occurred at approximately 107 °C (Figure 7). Previous investigations have reported the melting temperatures for virgin and recycled LDPE as between 102 and 105 °C, suggesting that the increase in the melting temperature of LDPE-Al may have been due to the presence of the aluminum particles and the fique fibers [12,26,27,28]. These may have acted as nucleation points that increased the crystallinity of the composite, increasing the energy that is required to melt the material.

Additionally, it was observed that the melt enthalpy of the materials that were reinforced with fique fibers decreased considerably with respect to the LDPE-Al (Table 2), which in turn was lower than the reference LDPE (98–115 J/g) [12,28]. This could be attributed to the increased proportion of aluminum and fique fibers, which do not melt under the test conditions, and which are not miscible with the polyethylene phase [29].

The percentage of crystallinity estimated for the polyethylene phase within the LDPE-Al matrix (44.53%) was higher than the 34% reported in previous DSC studies of LDPE [12]. This could be due to the presence of aluminum particles that acted as nucleation points and promoted the formation of the crystalline phase [28]. However, in fique fiber-reinforced materials, the crystalline phase content was maintained at 44.70%, despite the decrease in the aluminum content in the composite through the incorporation of fibrous material. This shows that fique fibers also promote the formation of crystalline phases in the polyethylene present in the composite. Some reports that were related to fiber-reinforced composites have found that the fibers act as nucleation points that increase the crystallinity of the polymer phase [19,30].

## 4. Conclusions

The presence of the dispersed phase of aluminum particles in the material increased the modulus by 592% and the crystallinity in the LDPE from 34% to 44.53%, because the aluminum particles act as nucleation points that promote the formation of the crystalline phase of the material.

One of the most important contributions of surface treating the fique fibers to the final properties of the composite was a reduction in the water absorption as compared to the composite reinforced with native fibers. This phenomenon was due to the improved fiber-matrix interface, which reduced the number and size of voids and/or cavities and the change in the hydrophilicity of the treated fiber.

The statistical analysis that was made on the results of the mechanical tests of the composites showed that the statistically significant variations in the tensile and flexural properties of the materials were mainly caused by the pre-impregnation of the fibers with polyethylene. This treatment promoted the wetting of the fibers with the polymer, possibly establishing other mechanisms of interfacial adhesion (i.e., interdiffusion and chemical bonding) besides mechanical adhesion, which predominated in the material due to the high aspect ratios of the fibers and their mat arrangement. Additionally, an increase in the fiber content promoted a statistically significant increase in the mechanical properties evaluated. Based on the properties of LDPE-Al, it was possible to increase the tensile and flexural strength by 218% and 322%, respectively, by using fibers that were treated with NaOH + silane + pre-impregnation. Finally, the tensile and flexural modulus improved with the incorporation of additional fibers, hardening the material.

## Figures and Tables

**Table 1:** Physical-mechanical properties of the materials used in the preparation of the composites.

Material	Low-Density Polyethylene-Aluminum	Native Fique	Fique Treated with NaOH	Fique Treated with NaOH + Silane	Fique Treated with NaOH + Silane + Pre-Impregnation
Nomenclature	LDPE-Al	Fique-N	Fique-A	Fique-AS	Fique-ASP
Density (g/cm^3^)	1.1	1.10	1.37	1.25	1.17
Equivalent diameter (mm)	-	0.21 ± 0.05	0.17 ± 0.04	0.17 ± 0.04	0.17 ± 0.04
Average aspect ratio	-	247.37	302.34	302.34	302.34
Maximum tensile strength (MPa) *	10.78 ± 0.23	336.12 ± 27.90	365.97 ± 25.58	370.99 ± 23.47	373.58 ± 22.54
Young’s modulus (GPa) *	0.56 ± 0.05	5.44 ± 0.45	6.53 ± 0.46	7.16 ± 0.54	7.83 ± 0.51
Rupture strain (mm/mm) *	0.130 ± 0.009	0.093 ± 0.007	0.077 ± 0.005	0.075 ± 0.005	0.058 ± 0.002

* The values were determined according to standard test method for tensile properties of single textile fibers (ASTM D3822-07) with a length between the grips of 25.4 mm (1 in).

**Table 2:** Parameters obtained from the Differential Scanning Calorimetry (DSC) analysis of the LDPE-Al and composites with 30% fique fibers with different surface modifications.

Material	Cooling			Second Heating			
	*T*_c_		Δ*H*_c_	*T*_f_		Δ*H*_f_	XPhase LDPE
	Onset Point	Peak		Onset Point	Peak		
	°C		J/g	°C		J/g	%
LDPE-Al	102.36	97.58	84.61	94.88	106.91	90.01	44.53
30Fique-N	102.98	98.21	61.54	94.95	106.88	63.22	44.70
30Fique-A	103.22	97.90	38.17	93.75	106.66	64.72	49.09
30Fique-AS	102.96	97.78	45.55	94.30	106.46	62.05	45.60
30Fique-ASP	102.54	96.39	45.10	93.24	108.12	61.06	43.92
